# Data Mining and Systematic Pharmacology to Reveal the Mechanisms of Traditional Chinese Medicine in Recurrent Respiratory Tract Infections' Treatment

**DOI:** 10.1155/2020/8979713

**Published:** 2020-10-26

**Authors:** Changyong Luo, He Yu, Tao Yang, Chen Bai, Bing He, Yurou Yan, Tiegang Liu, Junhong Wang, Xiaohong Gu

**Affiliations:** ^1^Beijing University of Chinese Medicine, Beijing, China; ^2^Dongzhimen Hospital of Beijing University of Chinese Medicine, Beijing, China

## Abstract

Traditional Chinese medicine (TCM) was widely used in the treatment of recurrent respiratory tract infections (RRTIs) in East Asia, but its mechanism was not clear because of its complex prescription rules. This research prospectively collected 100 prescriptions of RRTI children treated with TCM. The characteristics of TCM in prescriptions were described and analyzed, and the rules of prescriptions were analyzed by hierarchical clustering and association rules. The results showed that the principle of RRTI was to pay equal attention to cold and mild, and six new meaningful prescriptions were obtained. Among them, the new prescription composed of Astragali Radix (Huangqi), Atractylodis Macrocephalae Rhizoma (Baizhu), Saposhnikoviae Radix (Fangfeng), Angelicae Sinensis Radix (Danggui), and Paeoniae Radix Rubra (Chishao) was an important method to treat RRTI. In order to explore the mechanism of the new prescription, the research obtained the action target of each herb of the core prescription on Integrative Pharmacology-based Research Platform of Traditional Chinese Medicine, TCMIP v2.0. The target genes were enriched by Metascape, and 93 Kyoto Encyclopedia of Genes and Genomes (KEGG) pathways were obtained. According to the classification and statistics of KEGG type, it was found that the new prescription mainly intervened in the metabolic pathway dominated by amino acid metabolism. In addition, there were also many interventions in the nervous system-, endocrine system-, and digestive system-related pathways. This study summarized the prescription rule of TCM in the treatment of RRTI, analyzed the mechanism of supplementing deficiency, and provided a new idea for the treatment of RRTI.

## 1. Introduction

Recurrent respiratory tract infections (RRTIs) are one of the most common diseases in children and adolescents [1]. Data from the World Health Organization showed that RRTI mostly occurs in children under 5 years old, accounting for 10%–30% of all pediatric respiratory infections. The incidence of RRTI rises every year. In Southeast Asia and Africa, the mortality rate was as high as 23%, affecting about 2 million people [2, 3]. It was difficult to remove pathogens of children, which leaded to an increased tendency of repeated infection [4]. At the same time, it could increase the risk of asthma, myocarditis, nephritis, and other diseases [5, 6]. Children with immune deficiency of RRTI could be treated by antibody replacement therapy, but the cost was huge. It was estimated that the annual cost was about 20,000–30,000 euros per patient [7]. RRTI brought psychological and physiological pressures to children and caused heavy burdens to their families and society [8]. Therefore, developing safe, effective, and affordable medicine for RRTI was a matter of great urgency.

After more than 2000 years of development, traditional Chinese medicine (TCM) has formed a complete health care system from diagnosis to prognosis, which has accumulated valuable experience for clinical application and medical research. The efficacy of TCM has been recognized by more and more people and countries. Researchers from many countries around the world have studied the efficacy of TCM in relieving symptoms [9, 10]. According to the World Health Organization, about 4 billion people around the world used herbs to treat diseases [11]. Studies showed that TCM could reduce the incidence of RRTI and significantly improve the clinical symptoms of RRTI [12]. In clinical practice, experienced doctors could flexibly prescribe according to the principle of compatibility of TCM and illness, but to a certain extent, it could also bring considerable difficulties to clinical research [13]. The effective treatment of RRTI with TCM has always been the focus of many researchers and pediatric experts. The research on the basic treatment principle, prescription compatibility, and the mechanism of prescription action of TCM has caused widespread concern. Therefore, the analysis of the therapeutic prescription and mechanism of RRTI would help to improve the clinical efficacy of RRTI and increase our understanding of the pathogenesis of RRTI.

Data mining has been used to analyze the prescription patterns of TCM from clinical data and find the potential relationship between herbs and diseases. New prescription and core prescription were discovered based on the retrospective analysis of data of chronic kidney patients as well as frequency analysis and correlation evaluation of Chinese medicine prescriptions of chronic kidney diseases [13]. The researchers collected clinical data and used frequency analysis and association rule learning to explore the treatment of insomnia and depression with TCM. It showed TCM prescribing patterns in patients with sleep disorders and depression [14]. In terms of exploring the mechanism of action of TCM, systematic pharmacology provided a new research approach for the theory of the “multicomponent and multiobjective network” of TCM [15]. It integrated phytochemistry, pharmacology, and bioinformatics, effectively bridged the gap between Western medicine and traditional medicine, and also promoted the research on the mechanism of synergistic action between various herbs in prescriptions [16]. There was growing evidence that systematic pharmacology has been used to explore the complex mechanism of action of Chinese medicine prescriptions [17–19]. By analyzing the compound targets and the enrichment pathway of the Chinese medicine prescription, the mechanism of core prescription could be learned more deeply.

**Figure 1:**
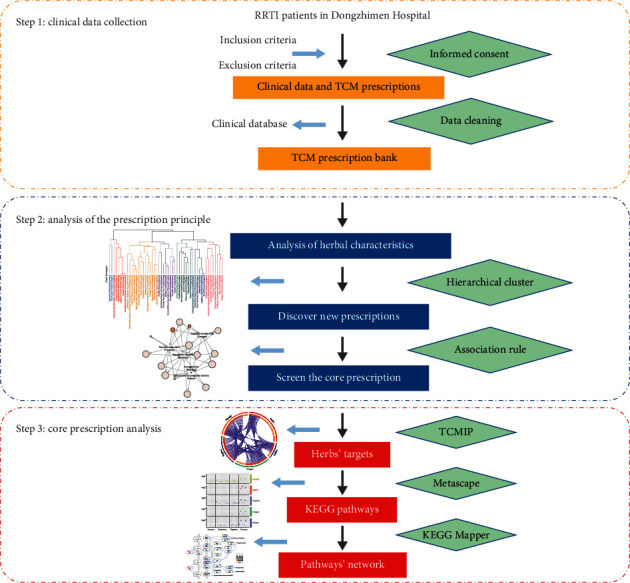
Workflow of the approach. Clinical cases of children with RRTI were prospectively collected. The main therapeutic principles, new meaningful prescriptions, and core prescriptions of RRTI were determined. Then, core prescriptions were analyzed by Metascape.

This study proposed a comprehensive method based on data mining and systematic pharmacology to analyze the rule of TCM prescription and reveal its mechanism. Clinical cases of children with RRTI were prospectively collected. These cases were prescribed by the chief physician of pediatrics from Dongzhimen Hospital, Beijing University of TCM. Then, the prescriptions of RRTI were analyzed by descriptive statistics, hierarchical clustering, and association rules to explore the medication rule of Chinese medicine prescriptions, and explore the core prescription, then retrieve the important targets from the database, and use Metascape to analyze Kyoto Encyclopedia of Genes and Genomes (KEGG) pathways. Workflow is shown in Figure 1. The purpose was to determine the main therapeutic principles, new meaningful prescriptions, and core prescriptions of RRTI through modern data evaluation, and the mechanism of core prescriptions was analyzed. It was concerned that this result could improve the understanding of the pathogenesis of RRTI and reveal the biological basis of the targets, so as to promote the research of TCM and the development of RRTI therapeutic medicine in the future.

## 2. Materials and Methods

### 2.1. Clinical Data Collection

From July 2019 to January 2020, clinical information and Chinese medicine prescriptions of children suffering from RRTI were collected prospectively from Dongzhimen Hospital of Beijing University of Traditional Chinese Medicine. RRTI diagnostic criteria referred to the Clinical Concept and Principles of Management of RRTI (Table 1), which was revised by the respiratory group of the Pediatric Society of Chinese Medical Association and the editorial committee of *Chinese Journal of Pediatrics*. Diagnostic criteria : (1) the interval between the two infections should be more than 7 days; (2) if the number of infections of the upper respiratory tract was insufficient, the number of lower respiratory tract infections could be added; otherwise, it could not be added; (3) the frequency should be determined by continuous observation for more than one year; and (4) repeated pneumonia should be in accordance with the signs of pneumonia and imaging examination, and between the two diagnoses, the signs and imaging changes should completely disappear.

  Inclusion criteria: (1) they should meet the diagnostic criteria of children's RRTI; (2) the age ranged from 1 to 17 years old; (3) it should be at least one week after the recovery of the acute infection; and (4) sign informed consent with the legal guardian or the tested child.
  Exclusion criteria: (1) children with severe primary respiratory tract infections such as primary immunodeficiency and acquired immunodeficiency syndrome (AIDS). (2) There were serious primary diseases such as heart, liver, kidney, digestive system, and hematopoietic system. (3) Participants in other clinical trials.

This study was reviewed by the Ethics Committee of Beijing University of TCM, with ethical batch number 2019BZHYLL0204, which was strictly implemented in accordance with the ethical system.

### 2.2. Data Collection and Analysis

#### 2.2.1. Data Collection

The information of patients' age, gender, diagnosis, and Chinese medicine prescriptions was collected.

#### 2.2.2. Data Entry and Cleaning

All data were entered into Microsoft Excel 2016, and the RRTI database of TCM was established. It included the patient's initials, hospital registration number, gender, age, diagnosis, and prescriptions, standardizing herbs as the official name according to the Chinese medicine classification of *People's Republic of China Pharmacopoeia (2015 Edition)* and *Chinese Medicine Dictionary* in the process of herbs' entry [20, 21]. The terminology used for herbs was standardized based on their official name, and descriptions were made based on the herbal properties. Herbs that were not included in Pharmacopoeia and textbooks, such as Stevia Rebaudiana (Tianyeju), had a unified name to ensure the order of the data. Two researchers completed the data entry separately.

### 2.3. Prescription Analysis

Through the descriptive analysis of the dosage, herbal properties, and taste of all prescriptions, the overall effect of prescriptions could be summarized and analyzed. Hierarchical clustering was applied to discover new prescriptions. Finally, this study used association rules to screen core prescriptions with strong relevance. GraphPad Prism 7 software was used to calculate the relationship between dosage, properties, and taste. RStudio version 3.5.1 was used for description analysis, hierarchical clustering, association rule analysis, and visual display.

#### 2.3.1. Descriptive Analysis: Dosage, Herbal Properties, and Taste

The characteristics of herbs were analyzed, including properties and taste. There are five items of herbal properties: cold, hot, warm, cool, and mild, and there are seven items of herbal taste: sour, bitter, sweet, pungent, salty, astringent, and light.

#### 2.3.2. Cluster Analysis to Discover New Prescriptions

Compatibility of TCM referred to the combination of two or more kinds of herbs according to clinical needs and the nature and function of medicine. The hierarchical clustering algorithm was used to cluster each herb. According to the similarity measurement among the objects, stable and regular new categories could be obtained in many individualized prescriptions. *A* and *B* in the clustering algorithm formula represent different herbs. Euclidean method was used to calculate the similarity between herbs, and cluster analysis was carried out for the top 50 herbs.

(1) $$\begin{array}{c} d\left(A,B\right)=\sqrt{\overset{n}{\underset{k=1}{\sum }}{\left(A_{k}-B_{k}\right)}^{2}}. \end{array}$$

#### 2.3.3. Association Rules to Screen the Core Prescription

Apriori algorithm was a frequent itemset algorithm to form association rules. It was used to analyze the clear rules of TCM in RRTI treatment and obtain the core herbs. In the data, each herb was treated as an itemset, and each prescription was treated as a transaction. Find out the frequent itemset in prescriptions, mining association rules between herbs, and filter the top 15 rules based on “support” to get the core prescription.. *A* and *B* in the association rule formula are frequent itemset herbs of one or several herbs. The correlation coefficient between herbs can be obtained by calculation. The support degree is set to 0.25, and the confidence degree is set to 0.8:

(2) $$\begin{array}{c} \text{support }\left(A=>B\right)=\frac{P\left(A\cup B\right)}{P\left(I\right)}, \end{array}$$

(3) $$\begin{array}{c} \text{confidence }\left(A=>B\right)=\frac{P\left(A\cup B\right)}{P\left(A\right)}, \end{array}$$

(4) $$\begin{array}{c} \text{lift }\left(A=>B\right)=\frac{P\left(A\cup B\right)}{P\left(A\right)P\left(B\right)}. \end{array}$$

### 2.4. Chemical Composition and Targets of the Core Prescription (CP)

Integrative Pharmacology-based Research Platform of Traditional Chinese Medicine, TCMIP v2.0 (http://www.tcmip.cn/TCMIP/index.php/Home/Login/login.html), was developed by the Chinese Academy of Chinese Medical Science. It mainly included five database resources from the Encyclopedia of Traditional Chinese Medicine (ETCM), “database of TCM prescriptions,” “database of TCM ingredients,” “database of TCM targets,” and “database of disease-related molecules.” [22] In this study, TCMIP was used to obtain the chemical components and molecular targets of the core prescription.

### 2.5. Functional Annotation and Enrichment Analysis

The obtained targets were enriched and analyzed with Metascape (http://metascape.org/gp/index.html#/main/step1) for the KEGG pathway. Upload the lists of genes and select H. sapiens species for analysis.. KEGG pathway analysis was carried out with the analysis mode of custom analysis [23, 24]. Then, only terms with both −log (*P* value) > 5 and more than 5% targets falling into the category were retained. Using ggplot2 of RStudio, the remaining terms were drawn into bubble charts. Finally, the bubble charts of each herb were combined.

### 2.6. Construction of the Core Prescription Access Network

To better elaborate the holistic mechanism of the CP, all targets of the CP were submitted to an online tool KEGG Mapper—Search and Color Pathway. Maps related to “Nervous” were reserved. The network was compiled by multiple pathways which were integrated and overlapped according to cross-talk targets. Network was drawn with Adobe Illustrator 2015CC, where intermediate genes were hidden for better display.

## 3. Results

### 3.1. Patient Characteristics

100 prescriptions of 68 children with RRTI were analyzed, with an average of 1.47 visits. 41 were male, 27 were female, and M : F ratio was 1.52. The average age was 5.27 ± 2.76. In addition to RRTI, 16 other diagnoses occurred. The frequency was 69% for pharyngitis, 47% for allergic rhinitis, 15% for hyperhidrosis, and 12% for dyspepsia.

### 3.2. Analysis of Herbal Characteristics

**Figure 2:**
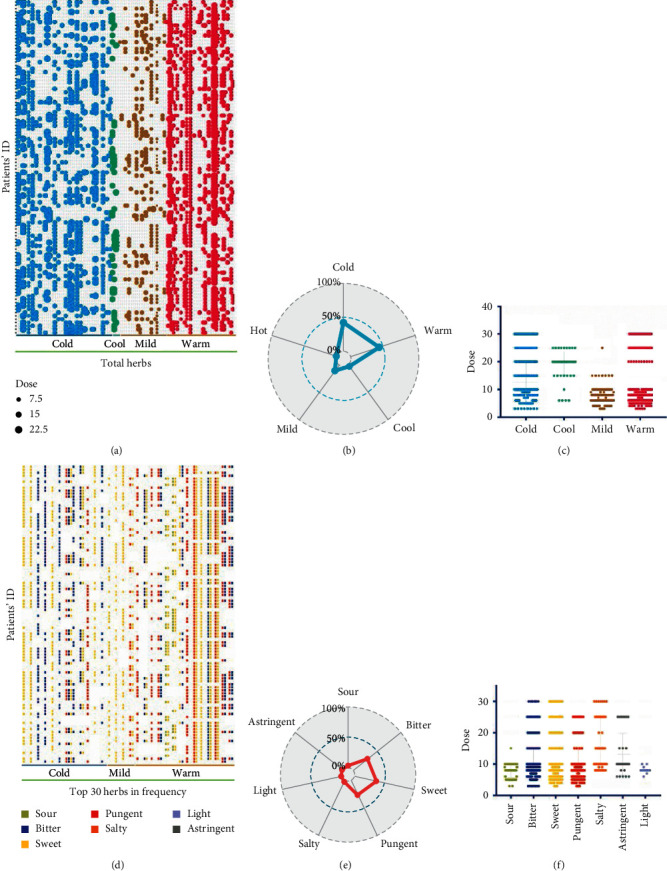
In (a), the *y*-axis was the patients' name code, and the *x*-axis was the herb. Herbal properties were divided into cold, cool, mild, and warm. The bubble size in the figure represented the dosage. The larger the bubble was, the larger the dosage was. Color corresponded to the *x*-axis property. (b) Radar chart showed the total frequency of each property in all prescriptions. (c) The dosage specification of different herbs. In (d), the *y*-axis was the patients' name code, and the *x*-axis was the herb. They were divided into cold, cool, mild, and warm according to the properties of herbs. Color of the box in the figure represented the taste of herbs. Radar chart (e) was the sum of the taste frequency of each herb in all prescriptions. (f) The corresponding dosage of different herbs.

In 100 prescriptions, there were 116 kinds of herbs, each prescription contained 21.46 herbs on average, and the total dosage of a single prescription was 230.14 g on average. Firstly, the properties of these herbs were analyzed. All the herbs, dosage, and properties are shown in Figure 2(a). Herbs of cold nature accounted for the greatest proportion followed by the herbs of warm nature; radar chart showed that the proportion of herbs of warm and cold nature was basically the same (Figure 2(b)). The dosage of herbs of cold nature was similar to that of herbs of warm nature. Based on the figures, the number of herbs with cold properties was found to be the most abundant, whereas herbs with warm properties were found to appear the most frequent in prescriptions. This meant there were fewer kinds of herbs with warm properties, but they appeared more frequently, whereas there were more kinds of herbs with cold properties, but they appeared less frequently in prescriptions. Overall, the total frequency of cold and warm drugs was similar.

Then, this study analyzed the taste of these herbs. The top 30 herbs and their taste are shown in Figure 2(d). The cold herbs were mainly bitter cold and sweet cold, and the warm herbs were mainly sweet warm and pungent warm; the flavor of sweet took the biggest part of the herbs followed by pungent and bitter (Figure 2(e)). The dosage of most herbs was about 10 g, and some of them were 20 g and 30 g, among which the dosage of bitter, sweet, and pungent herbs was similar (Figure 2(f)).

Table 2 shows the frequency, herbal properties, and taste of the top 30 herbs. Among them, the frequency of Saposhnikoviae Radix (Fangfeng) was the highest, 94 times, and the frequency of Astragali Radix (Huangqi), Atractylodis Macrocephalae Rhizoma (Baizhu), and Angelicae Sinensis Radix (Danggui) was 83 times. The frequency of other herbs in the descending order was as follows: Mori Cortex (Sangbaipi), Cicadae Periostracum (Chantui), Paeoniae Radix Rubra (Chishao), Fritillariae Thunbergii Bulbus (Zhebeimu), Cynanchi Paniculati Radix et Rhizoma (Xuchangqing), Imperatae Rhizoma (Baimaogen), Stevia Rebaudiana (Tianyeju), and Scutellariae Radix (Huangqin). The results showed that these herbs were commonly used in RRTI.

### 3.3. Hierarchical Cluster Analysis

**Figure 3:**
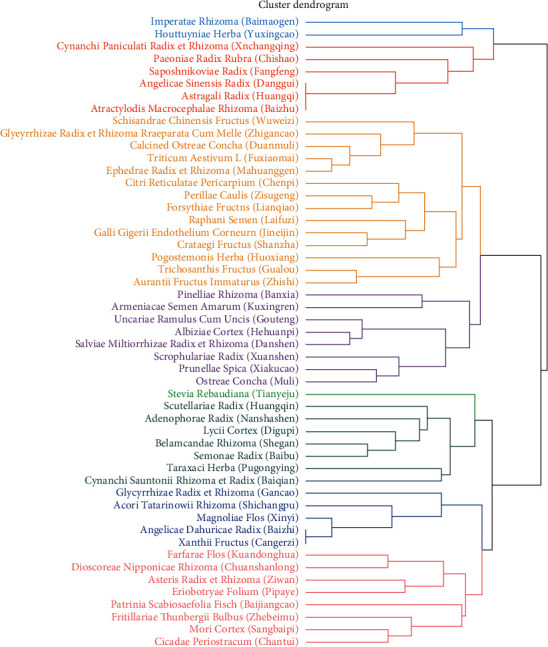
New prescription analysis: the results of the cluster tree showed that 50 herbs could be divided into 8 groups, which were labeled with different colors.

The hierarchical clustering method was used to classify herbs based on the relationship between herbs, which was used to determine the combination rule of different TCM treatment methods. The advantage of this method was that potential new prescriptions for RRTI could be found. In this study, core herbs with the frequency of top 50 were analyzed. The herbs were divided into 8 modules, and 6 new effective prescriptions were obtained according to the theory of TCM (Figure 3).

### 3.4. Association Rule Analysis

**Figure 4:**
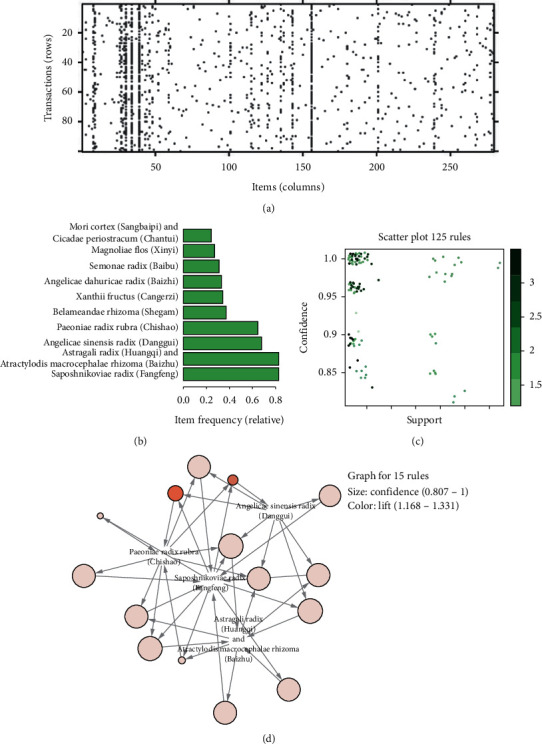
Core prescription analysis: (a) frequent items' matrix; *X*-axis stood for items, and *Y*-axis stood for transactions. (b) The top 10 frequent itemsets. (c) The details of all 125 rules, with *X*-axis as support and *Y*-axis as confidence. Color of the dot represented the lift in value, and the darker the color, the higher the lift. (d) The network diagram of association rules in the first 15 support levels. Size indicated conﬁdence (0.807–1), and color indicated lift (1.168–1.331).

We used the Apriori algorithm to analyze the association rules of the herbs in all prescriptions. First, the data were transformed into transactions. There were 100 transactions and 279 frequent itemsets (Figure 4(a)). The top 10 frequent itemsets were “Saposhnikoviae Radix (Fangfeng),” “Astragali Radix (Huangqi) and Atractylodis Macrocephalae Rhizoma (Baizhu),” “Angelicae Sinensis Radix (Danggui),”“Paeoniae Radix Rubra (Chishao),” “Belamcandae Rhizoma (Shegan),” “Xanthii Fructus (Cangerzi),” “Angelicae Dahuricae Radix (Baizhi),” “Stemonae Radix (Baibu),” “Magnoliae Flos (Xinyi),” and “Mori Cortex (Sangbaipi) and Cicadae Periostracum (Chantui)” (Figure 4(b)). Set support = 0.25, confidence = 0.8, minlen = 2, and maxlen = 5. There were 125 rules (Figure 4(c)). The maximum lift value was 3.30, 6 rules tie for first place. There were 52 herb association patterns with a confidence level of 1. See Table S1 for specific association rules. The top 15 rules of “support” were selected for visual display (Figure 4(d)). It could be seen that Astragali Radix (Huangqi), Atractylodis Macrocephalae Rhizoma (Baizhu), Saposhnikoviae Radix (Fangfeng), Angelicae Sinensis Radix (Danggui), and Paeoniae Radix Rubra (Chishao) were the herbs with the strongest correlation and in the core position. At the same time, it was found that the core herb was highly overlapped with new prescription 1 and could become the core prescription.

### 3.5. Overall Targets of the CP for RRTI Treatment

**Figure 5:**
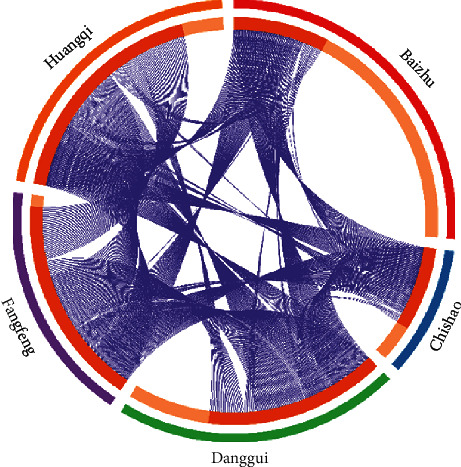
Herb target display: overlap between gene lists: the outer yellow ring represented Astragali Radix (Huangqi), red represented Atractylodis Macrocephalae Rhizoma (Baizhu), purple represented Saposhnikoviae Radix (Fangfeng), green represented Angelicae Sinensis Radix (Danggui), and blue represented Paeoniae Radix Rubra (Chishao). Purple curves connected the same genes. Inner circle represented the gene list. The same genes of multiple herbs were shown in dark orange. Genes that only appeared once was shown in light orange.

In order to fully understand the mechanism of the core prescription in the treatment of RRTI, this study collected and analyzed the known targets of the prescription. In the literature and TCMIP, five herb targets of the CP were obtained and visualized (Figure 5) [25].

The number of herb targets was 235 for Astragali Radix (Huangqi), 292 for Atractylodis Macrocephalae Rhizoma (Baizhu), 196 for Saposhnikoviae Radix (Fangfeng), 232 for Angelicae Sinensis Radix (Danggui), and 106 for Paeoniae Radix Rubra (Chishao).

### 3.6. Functional Analysis of Core Herbs

**Figure 6:**
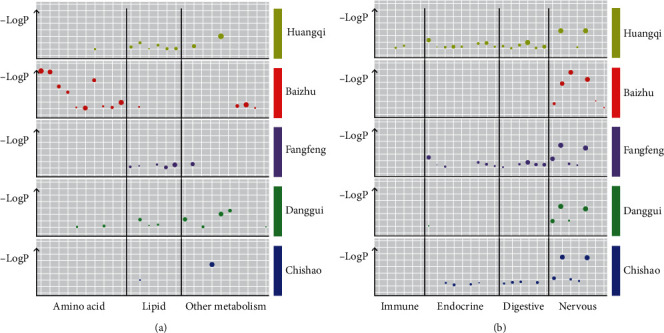
Kyoto Encyclopedia of Genes and Genomes (KEGG) enrichment analysis: bubble diagram of the KEGG enrichment pathway. Each bubble represented a KEGG pathway. The size of the bubble was related to the relative ratio of the target on each pathway to the total target, and the larger bubble represented the richer gene. −LogP showed the statistical significance of the *P* value. The larger the number was, the more significant the *P* value was. (a) The Amino acid metabolism, lipid metabolism, and other metabolism. (b) The immune system, endocrine system, digestive system, and nervous system pathways.

A total of 93 KEGG pathways were obtained. Analysis of KEGG type showed that 26 signaling pathways were metabolic-related pathways. It was proved that the combined effect of core prescription herbs played a large part in intervening the metabolism process of the body. Astragali Radix (Huangqi), Saposhnikoviae Radix (Fangfeng), and Angelicae Sinensis Radix (Danggui) were mainly responsible for the fatty acid metabolism pathway, while, Atractylodis Macrocephalae Rhizoma (Baizhu) was mainly responsible for the amino acid metabolism pathway (Figure 6(a)). In addition, it was also found that there were abundant pathways related to the nervous system, digestive system, endocrine system, and immune system. Astragali Radix (Huangqi) had a strong intervention role in the neuroendocrine-digestive system. Saposhnikoviae Radix (Fangfeng) and Paeoniae Radix Rubra (Chishao) could help Astragali Radix (Huangqi) to play a role in regulating the endocrine system of the body; five herbs in the core prescription had regulatory effects on multiple pathways of the nervous system, there were two immune system-related pathways involved, and only Astragalus had regulatory effect (Figure 6(b)).

### 3.7. Building the Pathway Network of the Nervous System of the Action of Core Herbs

**Figure 7:**
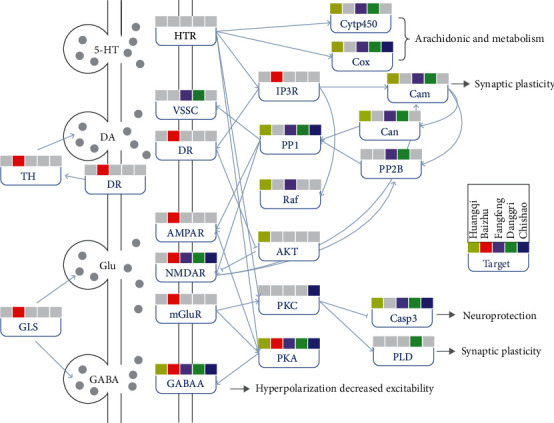
Building neural system pathway network: the neural system pathway network showed the regulation of core herbs on many neurotransmitters. The gene name was shown in rectangle. The gray dots represented neurotransmitters/hormones. The marker above each gene showed the targeting patterns of five herbs. In order to better illustrate the research problem, the middle gene was omitted and indicated by dotted arrows.

There were seven pathways belonging to the nervous system, which were, namely, long-term potentiation, retrograde endocannabinoid signaling, glutamatergic synapse, serotonergic synapse, GABAergic synapse, dopaminergic synapse, and long-term depression. Astragali Radix (Huangqi) has a wide range of interventions in the middle and lower reaches of the nervous system pathway, such as Raf, PP1, AKT, PKA, Casp3, Cam, Can, Cox, and Cytp450. Atractylodis Macrocephalae Rhizoma (Baizhu) has a unique intervening effect on the protrusion anterior membrane and protrusion posterior membrane. GABAA, NMDAR, PKA, Cox, and CASP3 were the targets of most herbs. Through the intervention of multiple pathways, it could regulate the functions of neuroprotection, hyperpolarization decreased efficiency, arachidonic acid metabolism, and synaptic plasticity (Figure 7). See Table S2 for specific pathways and genes.

## 4. Discussion

The nature and function of TCM formed the fundamental basis in analyzing Chinese medicine and its clinical applications. In this study, a comprehensive descriptive analysis was used to analyze the herbs, prescriptions, dosage, herbal properties, and taste. Overall, the total frequency of cold herbs and warm herbs was similar. Cold herbs have the effect of clearing away heat, and warm herbs have the effect of supplementing benefits. This discovery reflected the concept of TCM to supplement missing substances and remove excess substances in the body to regulate homeostasis [26–28]. Further analysis found that the frequency of cold herbs was less than that of core warm herbs, but there are more kinds of cold herbs than core warm herbs. This was balanced with warm herbs, which did not have as many varieties but were used at a much higher frequency. This meant in RRTI treatment, according to the basic theories of TCM, it might be necessary to select different combinations of cold herbs according to the clinical situation to play the role of clearing away heat, while the choice of warm herbs would be chosen between several core herbs.

The pathogenesis of RRTI is complex. In the noninfectious stage, it is usually associated with pharyngitis, allergic rhinitis, hyperhidrosis, dyspepsia, and other symptoms. There are also different emphases when prescribing TCM treatments. In this study, we found the relationship between herbs by cluster analysis and obtained 6 effective new prescriptions based on the theory of TCM. Prescription 1 mainly supplements benefits; prescription 2 has the functions of clearing stomach heat and reducing hyperhidrosis; prescription 3 could relieve fidgety and reduce thick turbinate; prescription 4 has the functions of clearing the lung and the pharynx; prescription 5 could unblock the nose; and prescription 6 could relieve coughs. Many of these symptoms match those that are displayed by children suffering from RRTI in clinical data. We speculated that RRTI has a certain pathophysiological relationship with the frequently occurring combined diseases or symptoms. In the process of treating RRTI, comprehensive treatment of TCM may produce better clinical effect. Association rule analysis revealed that Astragali Radix (Huangqi), Atractylodis Macrocephalae Rhizoma (Baizhu), Saposhnikoviae Radix (Fangfeng), Angelicae Sinensis Radix (Danggui), and Paeoniae Radix Rubra (Chishao) were closely related, and they were the core herbs in the treatment of RRTI. These herbs belonged to new prescription 1 in the cluster analysis, which was an important treatment module for RRTI, and were considered to be the main herbs to play the role of tonic. The results of meta-analysis show that Astragali Radix (Huangqi), Atractylodis Macrocephalae Rhizoma (Baizhu), and Saposhnikoviae Radix (Fangfeng) could improve the total clinical effective rate of RRTIs in children and reduce the incidence of respiratory infection [12].

The mechanism of core prescription in the treatment of RRTI was studied by systematic pharmacology. Five kinds of herbs in the core prescription played a synergistic role and intervened RRTI through various ways. The whole prescription was mainly aimed at metabolism-related pathways, especially fatty acid metabolism- and amino acid metabolism-related pathways. In addition, it showed that the core prescription with warming and tonifying effects could intervene in the enrichment of related pathways such as the nervous system, endocrine system, and digestive system, which has been proved by previous research results [29–31]. The above results seemed to support the theory of TCM. In the core prescription, Astragali Radix (Huangqi) played a role in improving metabolism, digestion, and endocrine function of the body. Atractylodis Macrocephalae Rhizoma (Baizhu), Saposhnikoviae Radix (Fangfeng), Angelicae Sinensis Radix (Danggui), and Paeoniae Radix Rubra (Chishao) played a role in assisting Astragalus in different aspects, which to some extent reflected the law of compatibility of Chinese medicine prescriptions [32]. It was reported that astragaloside IV and Astragalus glycoprotein could regulate immunity and promote axon and neuroprotection. In this study, Astragali Radix (Huangqi) could widely interfere with the downstream targets of nervous system pathways.

Combined with data mining and systematic pharmacology, this research comprehensively analyzed the prescription patterns of traditional prescriptions to explain the mechanism of traditional prescriptions and provide useful new enlightenment for the treatment of RRTI. This study prospectively collected clinical data and prescriptions of patients to ensure the quality of research data, but to some extent, there were still limitations due to insufficient samples. Big data analysis can summarize the group symptoms and prescriptions of RRTI patients from a macroperspective, and the new prescriptions produced are meaningful for understanding the pathological mechanism of RRTI, but the new prescription is not suitable for everyone. The clinical prescription should be individualized on the basis of syndrome differentiation and treatment. The new prescription can provide effective treatment guidance for the most common clinical syndromes. For example, the patients with “deficiency of lung Qi and spleen Qi” and “gastrointestinal heat accumulation” can choose the combination of new prescriptions 1 and 2. It is beneficial for doctors to improve the clinical efficacy by adding or subtracting herbs in clinical manifestations. Through the analysis of the mechanism of the prescription of TCM in the treatment of RRTI, more information was also revealed about the pathogenesis of RRTI. It is generally believed that RRTI is closely related to the immune and inflammation system [33, 34], such as myeloid differentiation primary response gene-88 (MyD88) signaling pathways, receptor-interacting serine/threonine-protein kinase-2 (RIPK2) signaling pathways, cytokine-cytokine receptor interaction, transformation-related protein 53 (p53) signaling pathway, and focal adhesion. This study found that the core prescription only impacted two immune pathways, whereas it had higher impact on amino acid metabolism- and neuroendocrine digestion-related pathways. On the one hand, it was suggested that the design of herbs for RRTI treatment in the future could cover not only the immune system but also the metabolism and neuroendocrine-digestive system of the body to obtain better clinical effect. On the other hand, this research showed that the heat-clearing herbs created the biggest impact through the immune system/inflammation modulation within the heat syndrome network [35, 36]. In the future, several other new prescriptions with heat-clearing mechanism as the main role will be studied to obtain more evidence. The safety and effectiveness of the core prescription still need to be further evaluated through clinical trials, and its mechanism also needs to be further explored and verified by further experiments.

## 5. Conclusions

A new prescription of RRTI composed of Astragali Radix (Huangqi), Atractylodis Macrocephalae Rhizoma (Baizhu), Saposhnikoviae Radix (Fangfeng), Angelicae Sinensis Radix (Danggui), and Paeoniae Radix Rubra (Chishao) has been extracted. It mainly intervenes in metabolism-, endocrine system-, and nervous system-related pathways. Research analysis and results showed that the method and software used in this study could effectively analyze the mechanism and law of traditional Chinese medicine prescription.

## Figures and Tables

**Table 1:** RRTI diagnostic criteria (frequency/year).

Age	URTI	LRTI	
		Recurrent bronchitis	Recurrent pneumonia
0–2	7	3	2
2–5	6	2	2
5–14	5	2	2

**Table 2:** Characteristics of top 30 herbs.

Herbs	Frequency	Properties	Taste
Saposhnikoviae Radix (Fangfeng)	94	Warm	Pungent, sweet
Astragali Radix (Huangqi)	83	Warm	Sweet
Atractylodis Macrocephalae Rhizoma (Baizhu)	83	Warm	Bitter, sweet
Angelicae Sinensis Radix (Danggui)	83	Warm	Sweet, pungent
Mori Cortex (Sangbaipi)	71	Cold	Sweet
Cicadae Periostracum (Chantui)	67	Cold	Sweet
Paeoniae Radix Rubra (Chaoshao)	64	Cold	Bitter
Fritillariae Thunbergii Bulbus (Zhebeimu)	60	Cold	Bitter
Cynanchi Paniculati Radix et Rhizoma (Xuchangqing)	55	Warm	Pungent
Imperatae Rhizoma (Baimaogen)	51	Cold	Sweet
Stevia Rebaudiana (Tianyeju)	51	Mild	Sweet
Scutellariae Radix (Huangqin)	50	Cold	Bitter
Asteris Radix et Rhizoma (Ziwan)	49	Warm	Pungent, bitter
Glycyrrhizae Radix et Rhizoma (Shenggancao)	47	Mild	Sweet
Farfarae Flos (Kuan Dong Hua)	44	Warm	Pungent, bitter
Eriobotryae Folium (Pipaye)	40	Cold	Sweet, pungent, bitter
Patrinia Scabiosaefolia Fisch (Baijiangcao)	40	Cold	Bitter
Taraxaci Herba (Pugongying)	39	Cold	Bitter, sweet
Houttuyniae Herba (Yuxingcao)	39	Cold	Pungent
Adenophorae Radix (Nanshashen)	38	Cold	Sweet
Gall1 gigerii endothelium corneum (Jineijin)	37	Mild	Sweet
Belamcandae Rhizoma (Shegan)	36	Cold	Bitter
Magnoliae Flos (Xinyi)	35	Warm	Sweet, bitter
Dioscoreae Nipponicae Rhizoma (Chuanshanlong)	35	Warm	Pungent
Angelicae Dahuricae Radix (Baizhi)	34	Warm	Pungent
Xanthii Fructus (Cangerzi)	34	Warm	Pungent, bitter
Crataegi Fructus (Shanzha)	33	Warm	Sour, sweet
Schisandrae Chinensis Fructus (Wuweizi)	31	Warm	Sweet, bitter
Stemonae Radix (Baibu)	31	Warm	Sour, sweet
Raphani Semen (Laifuzi)	30	Mild	Pungent, sweet

## Data Availability

All the data used to support the findings of this study are available from the corresponding author upon reasonable request.

## Supplementary Materials

Supplementary Materials Table S1: all 125 association rules are shown. Table S2: the pathways and genes of neural system-related pathway networks are shown.
